# The Treatment of Acute Pneumonia

**Published:** 1908-08-01

**Authors:** J. Walter Carr

**Affiliations:** Physician to the Royal Free Hospital and to the Victoria Hospital for Children, Chelsea.


### Hospital Clinics.

### THE TREATMENT OF ACUTE PNEUMONIA.

By J. WALTER CARR, M.D., F.R.C.P., Physician to the Royal Free Hospital and to the Victoria Hospital for Children, Chelsea.

(Abstract of a lecture delivered at the Polyclinic, June 24, 1908.)

I feel that I owe an apology for venturing to occupy your time over so hackneyed a topic as the treatment of pneumonia, on which everyone here may be as competent to offer an opinion as I am. Nevertheless, the subject of pneumonia, and especially its treatment, has excited the interest of physicians from the earliest days down to the present time. From the period of Hippocrates onwards this acute and often fatal malady has led to the utmost diversity of opinion, and of the acute febrile diseases there is probably none that has caused more discussion. It is the commonest of the more serious acute diseases, and is perhaps becoming more common. In all probability it is increasingly fatal; certainly that is so in the United States of America, and it may be also in this country: pneumonia is now almost, if not quite, as frequent a cause of death here as pulmonary tuberculosis.

I have nothing new to bring forward, no new theory or novel method of treatment, no vaccine to prevent, no serum or antitoxin to cure the disease. Nor have I even any suggestions of a heterodox character, but merely the old-fashioned measures. Note above all things in treating pneumonia the importance of attention to every detail, however small.

In children there is no disease in which the patient seems so ill, and yet is so likely to recover. This applies less to infants under twelve months of age, but even in them it is marvellous how small, comparatively, is the mortality; and in children over that age the rate is extraordinarily low. Hence I distrust profoundly any statistics of successful treatment based on experience derived from children or from cases including a large proportion of youthful patients.

On the other hand, there are cases in which, do what we may, the patients will inevitably die. When pneumonia is a terminal infection or occurs in what has been called the "spent" man, it is almost invariably fatal: and it is the deficient resistance of the patient rather than the virulence of the disease which chiefly contributes to this. One often hopes in such cases that if the patient can be kept alive until the crisis all may be well; but over and over again in these elderly individuals the crisis does not come when it is expected, and I believe that this is due to their having lost the power of forming the antitoxins by which the disease is brought to a natural termination. Not every old person is thus incapable of resistance; I have seen several patients over 70 who have survived attacks of acute pneumonia.

A third group contains those patients in whom the issue may be influenced by our treatment, and I think these cases constitute a very considerable percentage of the whole. It is among these that attention to detail in every shape and form is so important. For example, a good night may just turn the scale one way or the other. Therefore, first of all, the bed must be seen to. We sponge a patient to reduce his temperature, and the moment after this is completed may allow him to be covered with innumerable wraps. It is often with the greatest difficulty that the friends can be persuaded not to load the patient with blankets and even with an eiderdown quilt over them. Perhaps it is the rigor at the commencement, or the idea that the disease is due to a chill, which is responsible for this. I shall never forget the look of thankfulness which I received from a young patient when I insisted on the removal of a heavy quilt and blanket from her bed coverings. Keep the room cool also; 60° F. is an amply sufficient temperature, and 55° F. may possibly be better; after the crisis no doubt a rather higher temperature is desirable.

Let me say here that I distrust all methods of treatment that involve frequent disturbance of the patient. He should never be subjected to unnecessary examination: a little extension of the physical signs does not matter, and one can judge of the general condition without local examination of the chest.

Let him have plenty of fresh air; in America they have been treating these patients out of doors, and at any rate we should keep the windows widely open. I quite agree with Sir James Barr, who advises abundance of fresh air in preference to oxygen gas at intervals and a stuffy vitiated atmosphere the rest of the day. Certainly the patient should not be confined in anything like a tent, which is still often used with a steam kettle in the treatment of bronchopneumonia. Even a perfectly healthy man might be reduced to a serious state by twenty-four hours in such an atmosphere, and what the effect must be on a patient with pneumonia I can hardly conceive.

### Food.

On the question of food I have no theories to propound. In all acute febrile illnesses I think we are still too much under the influence of the reaction against the old methods of starvation and depletion. It is said that Graves ordered as the epitaph for his tombstone, "He fed fevers"; but it is possible to do almost as much harm by overfeeding patients as by starving them. A pneumonia patient has almost complete anorexia, and physiology teaches us that this is probably due to deficient gastric secretion, which in turn leads to deficiency of the other digestive juices. On the other hand we know that he is very thirsty: give him, therefore, plenty of water to drink, of which he is in need, but do not deceive him by substituting foods, such as milk or strong meat preparations. The constipation, concentrated urine, and dry skin all point to the need for water, and are indications for offering food freely diluted. There is a vague impression that a patient left unfed for twenty-four hours will die of starvation. Nothing of the sort; let your patient alone for twenty-four or even forty-eight hours, and he will be all the better for it. I do not say starve him absolutely, but do not attempt to overload his digestive organs with unnecessary food.

Another point: unless the patient becomes desperately ill, do let us make a distinction between night and day. In hospital one orders a certain diet, which is generally given at equal intervals throughout the twenty-four hours; and in private the same thing happens if the matter is left to the nurses. In fact, if the night nurse can get more food down than the day nurse, she is very pleased with herself; but this should not be allowed. The laws of health are not always altogether reversed in sickness; the patient has been accustomed, it may be for 30 or 40 years or more, to have no food for eight to twelve hours at night, and merely because he is ill there is no need to give him as much at night as in the day. Endless harm results from this sort of thing: the patient may require something once or twice or even thrice in the night, but he should certainly not be disturbed for it.

I am convinced that I have seen disasters follow this excessive feeding. I remember a few years ago going round a hospital ward in which I had a man with acute pneumonia. I noticed that he looked worse, and, as I usually do in such cases, put my hand on his abdomen and found it much distended. I asked how much food he had been given, and when I found out immediately ordered that he should have much less for some hours. He recovered satisfactorily; but he had been made worse by being given an excessive quantity of milk.

What shall we give? Milk is the only food suitable for a febrile patient which contains any material amount of fat. In addition, I believe in something from meat; we can increase the value of beef tea by adding Plasmon to it, which provides valuable and digestible nutriment in concentrated form. Somatose is expensive, but useful in the same manner for those who can afford it, or meat juice, which contains 5 per cent. of albumin, may be used. In one or other of these ways broth or beef tea can be made more digestible and palatable, and a variation provided from the everlasting recurrence of milk, which is so apt to cloy the patient. Sometimes a beaten-up egg with a little brandy may be ordered, but is not often necessary.

We do not use tea enough, I think, in the treatment of acute disease; the caffeine in it is a useful mild stimulant, and patients like to have tea to drink. Fruit juice—grape, orange, and so on—is also useful if there is no diarrhœa. Whey is not sufficiently appreciated in the treatment of pneumonia: it is highly digestible and yet contains a considerable amount of nourishment. Patients can be kept going very adequately indeed for a few days on whey if they cannot digest milk. Convalescents, as a rule, may be fed freely and fully without delay. Particularly is this so with young patients; let them have as much as they want, and recovery will be all the quicker.

### The Care of the Mouth.

We cannot be too careful to keep the mouth clean, for it may be a storehouse of pneumococci. If there are any false teeth take them out. It is extraordinary how quickly some nurses forget what they are taught in hospital, and they sometimes sadly fail in the care of the mouth, which should be wiped out with some antiseptic mouthwash after every feed, or at least after every milk feed.

I need hardly say, also, how important it is, in private practice more especially, to guard against bed-sores.

Closely associated with the relief of pain is the question of securing sleep. A patient with pneumonia who is sleepless will probably become delirious, and this complication makes the prognosis very much worse. There are few questions about which there has been more controversy than over the use of morphia in pneumonia. I used to order it a good deal, until I had a nasty experience with a man of sixty or so for whom I ordered a quarter of a grain of morphia to procure sleep. My house physician subsequently spent half the night performing artificial respiration. The patient died two or three days later, though not as the result of the treatment. Morphia or opium is distinctly dangerous in elderly patients, especially if there is any kidney mischief, and in the late stages of the disease. In younger people in the early stage a small dose may be safely given, such as five to ten grains of Dover's powder or a sixth of a grain of morphia subcutaneously.

To get a patient comfortably to sleep we can do a good deal by a combination of this sort; a good sponge down, preferably with iced water, about 10 p.m., followed by a drink of milk, half an ounce or an ounce of brandy, and thirty grains of bromide or ten of veronal. He will then, if left alone, probably go to sleep for a few hours. Give special directions that the patient is not to be disturbed for either food or medicine. At a later stage, if these measures do not succeed, we may give hyoscine in preference to morphia, but this drug also is not without risk. When a patient is delirious the margin between the dose of a hypnotic drug large enough to produce sleep and that likely to give rise to dangerous coma is a very narrow one.

Ought we to try to reduce temperature? I remember reading certain German lectures in which it was held that the high temperature is *the* cause of danger in pneumonia. It was pointed out that at the crisis—when the temperature falls—the symptoms improve at once. But there is another explanation of this coincidence, and the mere bringing down of fever does not remove the toxins from the circulation, and often does not improve the patient at all. Antipyretic drugs are a source of danger, and merely lower the vitality of the patient. In the very worst cases the temperature is not very high, for instance, in old people and in septic cases. We are simply deceiving ourselves if we do nothing but try to reduce the temperature. Sponging is extremely refreshing, as any of you will know who have had a very high temperature and been sponged for it. It may make the patient much more comfortable and inclined to sleep, but beyond that I do not think it does much good. Do not rely much upon it.

### Drugs.

As for drugs, the mild cases really require none. We may give a simple diaphoretic or diuretic mixture, such as twenty to forty grains of citrate of potash with some liquor ammoniæ acetatis in an ounce of water; or some dilute hydrochloric acid, with a grain or two of quinine, may help digestion a little.

Most authorities are agreed that they now give less and less alcohol in acute pneumonia. Sometimes a few doses seem to rally a patient who is overwhelmed, as it were, at the very outset of the illness, and give him time to recover from the sudden disturbance to the system. If he becomes desperately ill, alcohol certainly provides a readily oxydisable food which is absorbed without digestion directly from the stomach, whereas water is not, but must pass on into the intestine. At the time of the crisis, also, when collapse sometimes threatens, the patient may be saved by a few doses of alcohol. But large doses day after day do little good, and may do harm.

Strychnine is very popular nowadays; it is preferably given hypodermically, and may be especially useful when combined with a minim or two of liquor atropinæ. Similarly ammonium carbonate is a valuable stimulant, and less likely than alcohol to be followed by subsequent reaction. Another drug I have tried is adrenalin chloride subcutaneously, but I have found little good from it. Personally, I think digitalis is of no use for its action on the heart in acute pneumonia, though I know that some physicians think otherwise. This may be because it takes two or three days before it begins to act; in acute pneumonia we cannot afford to wait that length of time. A little time ago Sir Lauder Brunton recommended ten or fifteen grain doses of calcium chloride for heart failure in pneumonia, and I have tried it with some satisfaction. I have no theories about it, but I believe it may do good.

Lastly, in a few cases venesection may be useful to relieve the over-strained right ventricle. Rapidly increasing dulness to the right of the steruum, with a vigorous apex beat but a feeble pulse, and cyanosis are the indications for it. With the cyanosis there is often a peculiar restless dyspnœa. Eight, ten, or fifteen ounces of blood withdrawn by timely venesection may just tide the patient over the danger of heart failure.

Two old aphorisms in conclusion. 1. In pneumonia, as in all acute febrile illnesses, treat the patient, not the disease; until we get some definite specific antidote it is a fatal mistake to use one routine form of treatment for pneumonia. 2. Be most careful not to do more harm than good; it is not right to give dangerous doses of powerful drugs on the principle that desperate ills need desperate remedies. Let us always beware lest to the toxæmia due to the pneumococcus we add the poisonous effects of too potent drugs.

